# Role of Tumor Necrosis Factor-α in the Human Systemic Endotoxin-Induced Transcriptome

**DOI:** 10.1371/journal.pone.0079051

**Published:** 2013-11-13

**Authors:** Brendon P. Scicluna, Cornelis van 't Veer, Max Nieuwdorp, Karen Felsmann, Britta Wlotzka, Erik S. G. Stroes, Tom van der Poll

**Affiliations:** 1 Center for Experimental Molecular Medicine and Center for Infection and Immunity, Academic Medical Center, University of Amsterdam, Amsterdam, The Netherlands; 2 Department of Vascular Medicine, Academic Medical Center, University of Amsterdam, Amsterdam, The Netherlands; 3 Department of Endocrinology and Metabolism, Academic Medical Center, University of Amsterdam, Amsterdam, The Netherlands; 4 Division of Infectious Diseases, Academic Medical Center, University of Amsterdam, Amsterdam, The Netherlands; 5 SIRS-Lab GmbH, Jena, Germany; Ohio State University, United States of America

## Abstract

TNFα has been implicated in the pathogenesis of various inflammatory diseases. Different strategies to inhibit TNFα in patients with sepsis and chronic inflammatory conditions have shown contrasting outcomes. Although TNFα inhibitors are widely used in clinical practice, the impact of TNFα antagonism on white blood cell gene expression profiles during acute inflammation in humans *in vivo* has not been assessed. We here leveraged the established model of human endotoxemia to examine the effect of the TNFα antagonist, etanercept, on the genome-wide transcriptional responses in circulating leukocytes induced by intravenous LPS administration in male subjects. Etanercept pre-treatment resulted in a markedly dampened transcriptional response to LPS. Gene co-expression network analysis revealed this LPS-induced transcriptome can be categorized as TNFα responsive and non-responsive modules. Highly significant TNFα responsive modules include NF-kB signaling, antiviral responses and T-cell mediated responses. Within these TNFα responsive modules we delineate fundamental genes involved in epigenetic modifications, transcriptional initiation and elongation. Thus, we provide comprehensive information about molecular pathways that might be targeted by therapeutic interventions that seek to inhibit TNFα activity during human inflammatory diseases.

## Introduction

Tumor necrosis factor (TNF) α is a pleiotropic proinflammatory cytokine that mediates both beneficial and detrimental biological processes [1], [2]. TNFα is rapidly released after trauma, infection or exposure to endotoxin (lipopolysaccharide, LPS) and is one of the most abundant mediators of inflammation at local tissue level [3]. TNFα has been implicated in the pathogenesis of several inflammatory diseases. Indeed, TNFα inhibitors have been shown to be an effective therapy for patients with rheumatoid arthritis, psoriasis, psoriatic arthritis, ankylosing spondylitis and inflammatory bowel disease [3]. In contrast, different strategies to inhibit TNFα in patients with sepsis revealed no overall benefit with regard to mortality reduction [4]. Sustained anti-TNFα therapy has been shown to heighten the risk of infections [5], [6], which can be considered a reflection of the delicate balance of innate immunity, where abundant activation may cause collateral damage (such as in sepsis and endotoxemia) while modest activation serves to protect the host against invading pathogens [7], [8]. The fundamental genomic responses that ensue as a consequence of TNFα antagonism *in vivo* still require exploration.

Pan-genomic expression profiling of human endotoxemia coupled with knowledge-based analysis has provided valuable insights into the transcriptional responses that activate and resolve systemic inflammation in a setting of a predictable recovery [9]. Intravenous injection of LPS into healthy humans induces changes in whole genome mRNA expression profiles in blood leukocytes that show strong resemblance to the “genomic storm” induced by burn trauma or sepsis in patients [10], [11]. A recently conducted systematic comparison of the whole blood leukocyte genomic response elicited by inflammatory diseases or intravenous LPS in humans with that in the corresponding experimental models in mice revealed that while the genomic responses to different acute inflammatory stresses are highly similar in humans, these responses are not reproduced in mouse models [11]. These data indicate that mouse experiments are less relevant for insight in which inflammatory pathways are responsive to TNFα inhibition. Although TNFα inhibitors are widely used in clinical practice, the impact of TNFα antagonism on white blood cell gene expression profiles during acute inflammation in humans *in vivo* has not been studied before. We here leveraged the established model of human endotoxemia to examine the effect of TNFα inhibition on the genome-wide transcriptional responses in circulating leukocytes induced by intravenous LPS administration. Our study provides a benchmark characterization of the transcriptional responses in acute inflammation mediated by TNFα in human males *in vivo*.

## Materials and Methods

### Ethics statement

The study was approved by the institutional review board of the Academic Medical Center, Amsterdam, and conducted according to the declaration of Helsinki. Written informed consent was obtained from all volunteers.

### Subjects and study design

Twenty-one young and healthy male Caucasian subjects were enrolled in this study and described elsewhere [12]. They were administered placebo (normal saline) (n = 13) or etanercept (n = 8; Enbrel® 50 mg; Pfizer, USA) intramuscularly 48 hours prior to receiving a bolus infusion of 1 ng/kg body weight *Escherichia coli* LPS (cat. # 1235503, lot G2B274; United States Pharmacopeial Convention Inc., Rockville, MD). After an overnight fast, the volunteers had a catheter inserted into the antecubital vein of each arm. Baseline blood samples were collected in EDTA tubes before LPS infusion in the antecubital vein of the contralateral arm (t = 0). Blood was withdrawn and collected in EDTA tubes 4 hours after LPS infusion (t = 4). Leukocyte counts and differentials were determined in whole blood by standard flow cytometry [12]. PAXgene blood was collected from 8 etanercept-treated and 8 placebo-treated subjects (total n = 16) for microarray analysis.

### Whole-blood leukocyte RNA preparation and genome-wide transcriptional profiling by microarray

Peripheral blood mRNA was obtained using the PAXgene™ tube and PAXgene™ RNA isolation system (PreAnalytiX™, Qiagen, Venlo, Netherlands) as described by the manufacturer. Total RNA yield and purity (260 nm∶280 nm) were determined spectrophotometrically (Nanodrop). The integrity of the re-suspended total RNA was determined using the RNA Nano Chip Kit on the Bioanalyzer 2100 and the 2100 Expert software (Agilent). To increase the sensitivity of our gene expression assay the overload of globin mRNA of whole blood samples was reduced by applying the human GLOBINclear kit (Ambion/Appied Biosystems). Synthesis, amplification and purification of anti-sense RNA was performed using 300 ng of enriched mRNA per sample and the Illumina TotalPrep RNA Amplification Kit (Ambion/Applied Biosystems) following the Illumina Sentrix Array Matrix expression protocol. A total of 750 ng biotinylated cRNA was hybridized onto the HumanHT-12v3 expression bead chips (Illumina) containing 48,803 probes derived from the National Center for Biotechnology Information (NCBI) Reference Sequence RefSeq (Build 36.2, Rel 22) and the UniGene (Build 99) databases targeting more than 25,000 annotated genes. The GenomeStudio® Data Analysis Software (Illumina) was used for data collection of the scanned Beadchips (BeadScan 3.5.31). Missing values were imputed by using the k-nearest-neighbor algorithm (k = 15) as previously described [13]. The raw scan and imputed data were read using the *lumi* package [14], available through Bioconductor [15]. This was carried out using the R statistical package (version 2.13.1; R Foundation for Statistical Computing, Vienna, Austria). Quality control checks of the raw non-normalized data included visualization of the similarity matrix and hierarchical clustering analysis [16]. Preparation of cRNA and chip hybridization was carried out at SIRS-Lab GmbH (Jena, Germany). After quality assessment one subject in the placebo group was removed from further analyses. Variance stabilizing normalization (*vsn*) [17] was applied. All subsequent analyses were performed on *vsn*-transformed intensity values. Filtering the non-expressed probes (detection p-value<0.05) yielded 18,419 probes for statistical analysis. Non-normalized and normalized data are available at the gene expression omnibus (GEO) with accession number GSE36177.

### Gene co-expression network analysis

The weighted gene co-expression network construction algorithm was used to build the systemic LPS-induced gene co-expression network using normalized expression data (n = 8168 transcripts, q<0.01). This was carried out using the R statistical package (version 2.13.1; R Foundation for Statistical Computing, Vienna, Austria) as described in detail by Langfelder and Horvath [18]. Briefly, the Pearson's correlation matrix of 8168 probes was transformed into an adjacency matrix by using a “soft” power function to ensure scale free network construction [18]. The adjacency matrix was further transformed into a topological overlap matrix to enable the identification of modules (clusters) of highly correlating genes by implementing the previously described dynamic tree cut algorithm [19]. These modules are composed of sets of genes with high “topological overlap” [20]. Thus, the topological overlap matrix enables the detection of not only a direct interaction between a pair of genes but also their indirect interactions with all other genes in the network. Each module represents a cluster of co-expressed genes with a distinct expression pattern from other identified modules. In order to define module hub genes we made use of the module *eigengene* concept, defined as the first principal component of the module expression matrix, and, the module membership measure, *k* [18], [21]. Hub genes were defined on the basis of a high correlation between gene significance (t statistic) and module membership [18], [21], [22]. Co-expression network visualization was achieved by means of the Cytoscape® software (www.cytoscape.org) version 2.8.3 [23].

### Biological pathway, interactome and cell-specific expression inference

Biological pathway analysis and interactome inference of each co-expression module was performed by using IPA (Ingenuity® systems, www.ingenuity.com). Gene significance (q-value) and log2 fold changes resultant from the paired analyses: 1. Placebo (t0) and LPS (t4); 2. Etanercept (t0) and LPS+Etanercept (t4), where incorporated. All analyses were performed on genes presenting false discovery rates <5% (q-value<0.05). Insight into the cell-specific patterns of systemic co-expression was gained by utilizing the co-expression atlas over-representation analysis available in the ToppGene bioinformatics suite (http://toppgene.cchmc.org/) [24].

### Quantitative real-time PCR

Total RNA was treated with RQ1 RNase-Free DNase (Promega, Leiden, The Netherlands) and reverse transcribed using oligo (dT) primer and Moloney murine leukemia virus reverse transcriptase (Invitrogen, Breda, The Netherlands) according to supplier recommendations. We used the LightCycler system (LC480, Roche Applied Science) to perform quantitative PCR analysis targeting transcripts encoded by *IL1RN*, *IRAK3*, *TNFAIP3*, *GPR84* and *MARCKS* genes. Results were normalized to *B2M* transcript. Primer pairs used were designed to avoid overlap with array probes (**Table S2 of File S1**).

### Statistical analysis

Pairwise analysis by Student *t* statistics was employed to assess differential gene expression in paired samples. Linear models ANOVA was employed to define endotoxin-induced transcripts influenced by etanercept treatment. Unless otherwise stated, a false-discovery-rate (FDR) corrected *p*-value (*q*-value) was used to define genome-wide significance.

## Results

### Systemic characteristics in response to LPS and TNFα antagonism

Twenty-one young healthy male subjects were challenged with an intravenous infusion of LPS (1 ng/kg body weight) as previously described [12]. Eight of these subjects were pre-treated intramuscularly with the TNFα antagonist etanercept (48 hours prior to LPS infusion); whereas the other thirteen received placebo by intramuscular injection. Etanercept treated subjects showed a strong rise in the circulating levels of soluble TNF receptor type II, providing evidence for effective administration [12]. Etanercept strongly inhibited LPS-induced systemic inflammatory responses known to be mediated by TNFα, including interleukin (IL)-6 and C-reactive protein release, indicating that etanercept effectively inhibited TNFα activity [12]. “Considering the sixteen subject samples used in the microarray analysis, no differences in cell counts were observed in the etanercept-treated group (n = 8) when compared to the placebo-treated group (n = 8) prior to LPS infusion (**Table 1**).”. Intravenous LPS induced a significant increase in total leukocyte counts, primarily caused by a rise in neutrophil numbers; monocyte and lymphocyte counts decreased after endotoxin infusion. Etanercept did not influence the LPS-induced changes in leukocyte counts or differentials (**Table 1**).

**Table 1:** White blood cell counts and differentials before and 4 hours after intravenous endotoxin administration in healthy subjects pretreated with etanercept or placebo.

	Baseline (t = 0)		Post Endotoxin (t = 4 hours)	
	*Placebo*	*Etanercept*	*Placebo*	*Etanercept*
Cell Type	Mean ±SEM (n = 8)	Mean ±SEM (n = 8)	Mean ±SEM (n = 8)	Mean ±SEM (n = 8)
WBC (×10^9^/L)	5.00±0.19	5.35±0.63	9.80±0.74***	10.64±0.98###
Neutrophils (%)	54.31±1.73	52.26±3.08	87.01±5.04***	82.83±1.66###
Eosinophils (%)	2.72±0.14	2.88±0.44	8.73±0.94***	8.73±0.72###
Basophils (%)	1.86±0.19	3.31±0.93	0.36±0.16***	1.51±0.67###
Lymphocytes (%)	34.44±1.76	36.66±3.17	9.39±3.96***	11.68±0.66###
Monocytes (%)	8.96±0.48	7.26±0.8	1.94±0.39***	3.69±0.77##

Sixteen healthy males received an intravenous injection with endotoxin (1 ng/kg) at t = 0. Subjects were pretreated (−48 hours) with etanercept (50 mg, n = 8) or placebo (n = 8). White blood cell counts (WBC) and differentials were determined directly before and 4 hours after endotoxin injection.

***p<0.001; paired student *t* test of placebo-treated baseline and endotoxin infusion groups.

## p<0.01,

### p<0.001; paired student *t* test of etanercept-treated baseline and endotoxin infusion groups. There were no differences between etanercept and placebo treated subjects at either t = 0 or t = 4 hours.

### Impact of TNFα inhibition on the genome-wide transcriptional response to intravenous LPS administration

**Figure 1:**
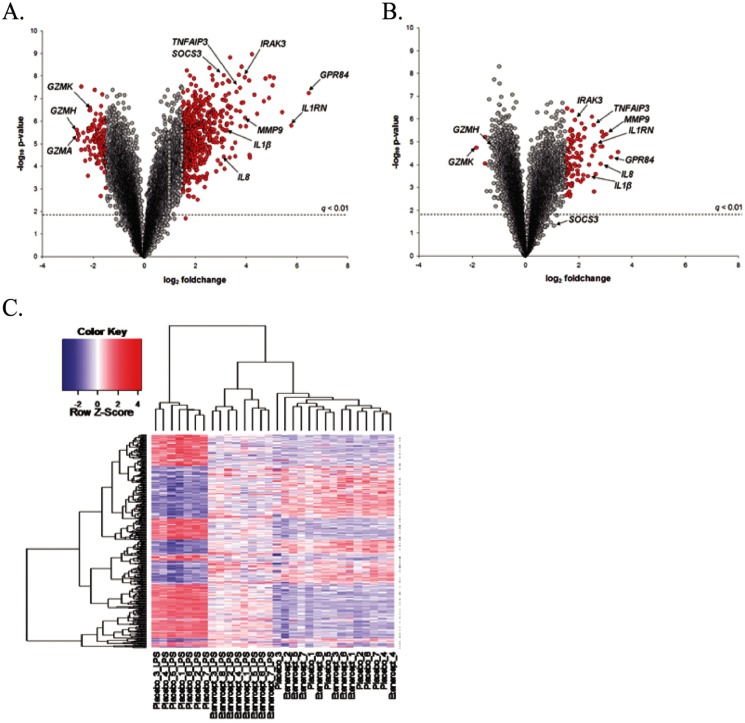
Genomic analysis of the systemic LPS-induced transcriptional response and impact of TNFα inhibition. **A**. Volcano plot analysis (integrating p-values and log2 foldchanges) for the LPS-induced response in subjects treated with placebo. **B**. Volcano plot analysis of the LPS-induced response in subjects treated with the TNFα antagonist etanercept. Red dots in panels A and B indicate probes that showed a fold-change ≥1.5 or ≤1.5. **C**. Unsupervised hierarchical clustering heatmap of the 4077 LPS-induced transcripts that were influenced by etanercept treatment as identified by ANOVA (q-value <0.05). Columns represent subject samples and rows represent transcripts. Red indicates increased gene expression, and blue indicates decreased gene expression.

Genome-wide transcriptional analysis was performed in whole blood leukocytes obtained before (t = 0) and 4 hours after LPS injection (t = 4) to study the impact of LPS challenge on gene expression. Considering a *q*-value threshold of 0.01, we detected 8168 probes that were differentially expressed. **Figure 1A** shows the volcano plot (integrating fold-changes and *t*-test criteria) for the LPS response in subjects treated with placebo. We detected 693 probes (*q*<0.01) with a fold change ≥1.5 or ≤−1.5. *GPR84*, *IL1RN*, *MMP9*, *IRAK3*, *TNFAIP3*, *SOCS3*, *IL1β* and *IL8* were among the highly induced transcripts (**Figure 1A**). Next, we assessed the transcriptional response to LPS challenge with TNFα inhibitor by comparing the signal intensities of probes obtained from etanercept-treated baseline (t = 0) and etanercept-treated LPS-challenged (t = 4 hours) samples. We detected 6583 probes that were significantly different under TNFα antagonism (*q*<0.01). Etanercept treatment resulted in a substantial effect on the LPS-induced transcriptional response (**Figure 1B**): only 106 probes (*q*<0.01) had a fold-change ≥1.5 or ≤−1.5. To assess the direct influence of TNFα inhibition on the LPS-induced transcriptional response we constructed and compared two linear models: 1. post-LPS response (t4) as a function of baseline gene expression (t0) and etanercept treated post-LPS response (t4); 2. the post-LPS response (t4) as a function of baseline gene expression (t0). Using an analysis of variance (ANOVA) we detected 4077 significant probes (*q*<0.05) that were responsive to TNFα inhibition. **Figure 1C** shows the unsupervised hierarchical clustering heat map of these 4077 probes. Of note, in the context of LPS-induced repression of gene expression, etanercept treament predominantly diminished the magnitude of those repressed genes. However, 2 significant transcripts (ANOVA q<0.05, foldchange ≥1.5), namely *CXCR4* and *KIAA0319L*, exhibited a directional change in their expression pattern, that is decreased by LPS while increased in expression post-etanercept treament. Interestingly, the LPS-inducible genes *TNF* and *TNFRSF1B* (*TNFR2*) were not significantly influenced by etanercept treatment (*q* = 0.18 and *q* = 0.17, respectively). However, *TNFRSF1A* (*TNFR1*) gene expression was significantly decreased (*q* = 0.0028) by etanercept. The complete list of those 4077 significant transcripts, their fold changes, *p*- and *q*-values, is tabulated in **Table S1**. Quantitative RT-PCR analysis on a few select transcripts was performed as a means of technical validation (**Figure S1 of File S1**). Of note, etanercept administration had no statistically significant effect on basal gene expression, that is between placebo (t0) and etanercept-treated (t0) samples.

### Identification of LPS-induced TNFα responsive and non-responsive co-expression modules

**Figure 2:**
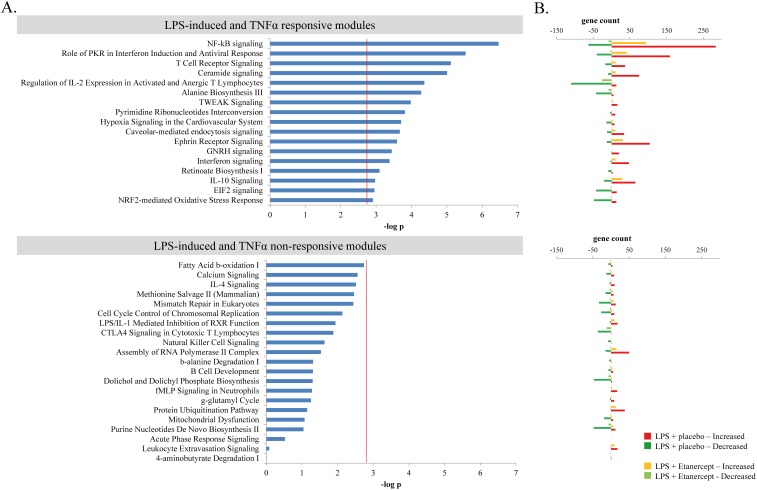
LPS-induced TNFα responsive and non-responsive transcriptional module delineation by weighted correlation analysis. Each transcriptional module, encompassing highly intercorrelating transcripts, was represented by its first principal component across all samples (module eigengene). **A**. Bar plot of module significance for the effect of etanercept on the LPS-response based on unpaired student *t* statistics of the module *eigengene* between post-LPS samples from placebo and etanercept-treated subjects. The red line denotes the multiple-test corrected significance threshold (−log_10_ *p* = 2.88). **B**. Bar plot denoting the upregulated and downregulated gene counts per IPA (ww.ingenuity.com) interactome pathway for both the LPS-challenged placebo-treated and LPS-challenged etanercept-treated samples.

In order to understand the relationship and organization of the LPS-induced transcriptome we applied the weighted gene co-expression network analysis approach [18], [25], [26] on the normalized expression data of the 8168 LPS-response transcripts (*q*<0.01). Considering the weighted Pearson's correlation matrix of these gene expression profiles 38 transcriptional modules, each encompassing more than 30 transcripts, were identified. **Table 2** lists the enriched canonical pathways per co-expression network module (cluster) as identified by Ingenuity pathway analysis (IPA). These biological pathways include NF-kB signaling, role of PKR in interferon induction and antiviral response, T cell receptor signaling, regulation of IL-2 expression in activated and anergic T lymphocytes, ephrin receptor signaling, ceramide signaling, IL-10 signaling, B cell development and natural killer cell signaling. Each module was summarized by its module *eigengene* (first principal component) [18], [25] followed by pair-wise analysis (*t* statistic) between LPS-challenged (t = 4 hours) placebo- and etanercept-treated samples. This analysis identified 17 modules significantly associated (Bonferroni corrected p-value threshold  = 0.0013) to TNFα antagonism (**Figure 2A**). LPS-induced and TNFα responsive modules include NF-kB signaling, role of PKR in interferon induction and antiviral response, T cell receptor signaling and regulation of IL-2 expression in activated and anergic T lymphocytes. LPS-induced and TNFα non-responsive modules include IL4 signaling, B cell development, natural killer cell signaling and LPS/IL-1 mediated inhibition of RXR function. Figure 2B illustrates the number of genes within their respective modules that are induced (log2 foldchange >0.5) or reduced (log2 foldchange <−0.5) in expression after LPS challenge, with or without etanercept treatment. In so doing we observed that among the TNFα responsive modules, TNFα antagonism heralds a decrease in expression of modules harboring genes predominantly involved in innate immune response reactions, such as NF-kB signaling and interferon signaling; whereas, in contrast, an increase in expression of modules comprised of genes involved in T cell responses, such as regulation of IL-2 expression in activated and anergic T lymphocytes (**Figure 2B**).

**Table 2:** Functional annotation and hub genes for the LPS-induced co-expression modules.

Canonical pathway	Module size	p-value	Hub gene	Hub gene functional signature
NF-kB signaling	829	3.4×10^−8^	*HIVEP1*	transcription factor activity
Role of PKR in Interferon Induction and Antiviral Response	578	8.8×10^−8^	*CBX7*	chromatin modification
T Cell Receptor Signaling	131	0.015	*PLEKHA1*	lipid binding
Ceramide signaling	221	7.0×10^−5^	*SRP54*	RNA binding
Regulation of IL-2 Expression in Activated and Anergic T Lymphocytes	218	0.001	*CD6*	receptor activity
Alanine Biosynthesis III	68	0.003	*GPR56*	receptor activity
TWEAK Signaling	61	3.4×10^−4^	*CTSH*	endopeptidase activity
Pyrimidine Ribonucleotides Interconversion	44	0.055	*POLR2G*	RNA polymerase activity
Hypoxia Signaling in the Cardiovascular System	89	0.011	*TTC13*	unknown
Caveolar-mediated endocytosis signaling	216	0.008	*MRFAP1L1*	unknown
Ephrin Receptor Signaling	247	1.6×10^−4^	*LIMK2*	kinase activity
GNRH signaling	63	4.2×10^−4^	*DNAJC5*	heat shock protein binding
Interferon signaling	97	1.7×10^−5^	*C6orf150*	DNA binding
Retinoate Biosynthesis I	99	0.006	*EIF2B5*	translation initiation activity
IL-10 Signaling	205	0.002	*ZBTB40*	DNA binding
EIF2 signaling	243	2.4×10^−24^	*RPS28P4*	unknown
NRF2-mediated Oxidative Stress Response	195	0.012	*APRT*	nucleotide binding
Fatty Acid b-oxidation I	48	0.002	*VPS36*	lipid binding
Calcium Signaling	64	0.003	*CD84*	cell adhesion
IL-4 Signaling	53	9.8×10^−4^	*N4BP2L1*	unknown
Methionine Salvage II (Mammalian)	60	0.012	*EP400*	chromatin modification
Mismatch Repair in Eukaryotes	131	2.0×10^−4^	*USP14*	endopeptidase activity
Cell Cycle Control of Chromosomal Replication	127	0.016	*POLR2H*	RNA polymerase activity
LPS/IL-1 Mediated Inhibition of RXR Function	60	8.7×10^−4^	*SLC27A1*	nucleotide binding
CTLA4 Signaling in Cytotoxic T Lymphocytes	100	0.001	*ITFG2*	unknown
Natural Killer Cell Signaling	54	5.1×10^−8^	*SH2D1B*	phosphotyrosine binding
Assembly of RNA Polymerase II Complex	213	0.003	*PAIP2*	translation repressor
b-alanine Degradation I	33	0.004	*CHKA*	cholinesterase activity
B Cell Development	72	5.8×10^−8^	*CD79B*	receptor activity
Dolichol and Dolichyl Phosphate Biosynthesis	225	1.8×10^−4^	*GLUD1*	oxidoreductase activity
fMLP Signaling in Neutrophils	49	0.006	*XAF1*	zinc ion binding
g-glutamyl Cycle	206	0.008	*RPL6P19*	RNA binding
Protein Ubiquitination Pathway	137	0.008	*RBM18*	RNA binding
Mitochondrial Dysfunction	143	9.3×10^−5^	*AKR1A1*	aldehyde reductase activity
Purine Nucleotides De Novo Biosynthesis II	211	2.5×10^−4^	*GOT2*	transaminase activity
Acute Phase Response Signaling	79	0.006	*GABPB2*	transcription factor activity
Leukocyte Extravasation Signaling	170	0.003	*VBP1*	unfolded protein binding
4-aminobutyrate Degradation I	99	0.017	*ALS2CR2*	kinase activity

LPS-induced transcriptome is organized into 38 co-expression network modules. Each module was analyzed for enrichment of biological pathways by IPA (Ingenuity® systems, www.ingenuity.com).

### LPS-induced and TNFα mediated co-expression module hub genes and in silico immune cell-specific enrichment

**Figure 3:**
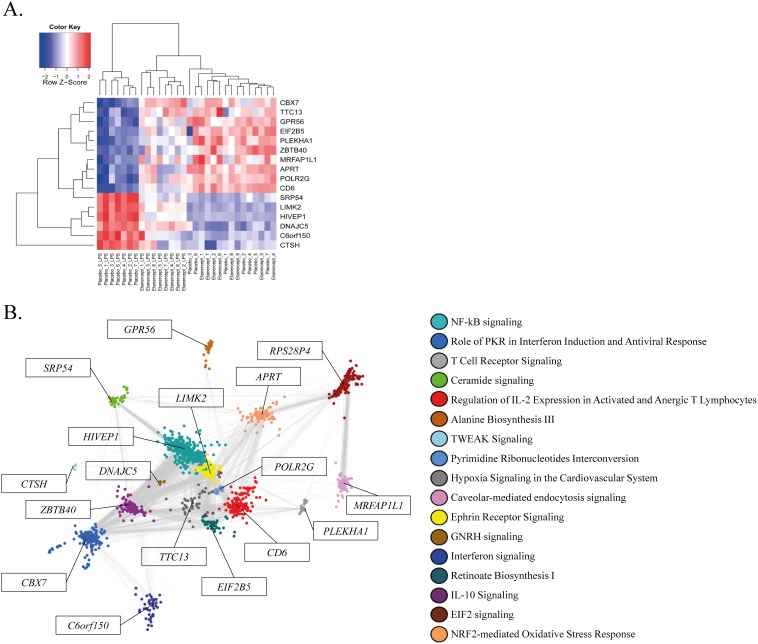
TNFα responsive module hub (driver) genes and co-expression network visualization. Genes within transcriptional modules can be categorized as peripheral or hubs on the basis of how correlated a gene is with all other genes in the network, defined as the genes' connectivity measure, **k**. High intramodulr connectivities denote highly important module genes oftentimes possessing transcriptional factor activity. **A**. Unsupervised hierarchical clustering heatmap plot of the TNFα responsive module hub genes. Red denotes high expression; blue denotes low expression. The relative importance of each module within the co-expression network can be highlighted by unsupervised visualizations of each genes' weighted correlation coefficient. This was implemented in the Cytoscape® platform **B**. TNFα responsive co-expression modules were visualized by an organic layout considering weighted correlation coefficients >0.1 (equivalent to correlation coefficient >0.9).

A fundamental property of biological networks is the emergence of a few greatly connected genes (nodes), often referred to as “hubs”, which suggests that these hub proteins possess a special biological role [21]. Genes within co-expression modules are interconnected by correlation and the relative importance of each gene within its respective module can be estimated by the connectivity value, *k*, and module membership, *k_MM_* [18], [22], [25]. The top module hub genes for the LPS-induced transcriptome influenced by TNFα antagonism are tabulated in **Table 3**. Functional signatures of these hub genes include epigenetic regulation of transcription, transcription factor activity and translation initiation/repression activity. Unsupervised hierarchical clustering of the hub gene expression patterns among LPS-induced TNFα responsive modules is shown in **Figure 3A**. This shows that the gene expression profiles of these LPS-induced and TNFα responsive module hub genes not only cluster according to experimental group as expected, but can be categorized into two groups of up-regulated and down-regulated transcripts. **Figure 3B** illustrates the Cytoscape γFiles organic visualization of the 17 LPS-induced and TNFα responsive transcriptional modules. In so doing, we show that NF-kB signaling, ephrin receptor signaling, role of PKR in interferon induction and antiviral response, regulation of IL-2 expression in activated and anergic T lymphocytes and IL10 signaling co-expression modules are highly connected. To better understand the cell-specific implications of these up- and down-regulated module hubs together with their respective module genes, we analyzed the clustered modules for enrichment in the coexpression atlas of the Immunological Genome Project (Immgen.org) [27] through the ToppGene bioinformatics suite [24]. TNFα-responsive down-regulated module hubs and peripheral genes were enriched for myeloid cells, including CD11b+ Ly6-G+ granulocytes (GSM854338, bonferroni p = 1.67×10^−24^) and B220− CD3− CD115+ Ly-6C/Glo CD43+ monocytes (GSM854332, bonferroni p = 9.11×10^−13^). TNFα responsive up-regulated module hubs and peripheral genes enriched for alpha beta T cells CD8+ CD45.1+ (GSM605894, bonferroni p = 1.71×10^−15^) and gamma delta T cells TCRd+ Vg2− CD44− (GSM476684, bonferroni p = 4.43×10^−11^). These findings further suggest TNFα antagonism decreases myeloid cell transcriptional events induced by LPS, whereas lymphoid cell transcriptional processes are increased.

**Table 3:** LPS-induced TNFα responsive module genes linked to transcriptional initiation, elongation and epigenetic regulation.

		*Epigenetic regulators*					
Functional group	Gene name	Module	kTotal	kWithin	kOut	*log2* FC LPS	*log2* FC LPS+Etan
DNA/RNA methylation	*DNMT1*	Regulation of IL-2 Expression in Activated and Anergic T Lymphocytes	45.43	13.52	31.9	−1.7	−0.9
	*RNMT*	Regulation of IL-2 Expression in Activated and Anergic T Lymphocytes	101.61	15.2	86.41	−1.2	−0.5
histone deacetylation	*HDAC1*	IL10 signaling	31.15	4.8	26.35	−0.6	−0.2
	*HDAC2*	NF-kB signaling	33.61	16.23	17.38	0.6	−0.03
	*HDAC3*	Role of PKR in Interferon Induction and Antiviral Response	30.08	8.33	21.76	−0.5	−0.1
histone acetylation	*HAT1*	Ephrin Receptor Signaling	41.06	8.44	32.61	0.3	0.09
histone-lysine N-methyltransferase	*SETD1B*	Interferon signaling	26.66	5.07	21.59	−0.3	0.06
chromobox/HP1 homologs	***CBX7***	Role of PKR in Interferon Induction and Antiviral Response	81.81	23.2	58.61	−0.8	0.03
	*CBX4*	GNRH signaling	0.26	0.15	0.11	0.4	0.1
	*CBX5*	Hypoxia Signaling in the Cardiovascular System	0.35	0.03	0.32	−0.3	−0.03
bromodomain	*BRD3*	Interferon signaling	55.95	4.79	51.16	−1.2	−0.3
	*BRD8*	IL-10 Signaling	1.53	0.38	1.16	0.4	0.1
Chromodomain/Helicase/DNA-Binding Domain	*CHD2*	NF-kB signaling	47.04	19.05	27.99	0.9	0.3
	*CHD4*	Caveolar-mediated endocytosis signaling	14.18	7.11	7.07	0.8	0.2
	*CHD1*	Role of PKR in Interferon Induction and Antiviral Response	2.4	0.83	1.56	1.1	0.5
chromatin insulator binding	*CTCF*	Interferon signaling	8.05	2.07	5.98	−0.6	−0.2

		*Transcription initiation factors*					
Functional group	Gene name	Module	kTotal	kWithin	kOut	*log2* FC LPS	*log2* FC LPS+Etan
TATA-binding protein	*TBP*	NF-kB signaling	36.75	12.71	24.03	−0.5	−0.01
TBP-class binding	*GTF2B*	IL-10 Signaling	85.48	18.1	67.38	1	0.5
	*GTF2A2*	NF-kB signaling	1.56	0.5	1.06	0.4	−0.03
RNA polymerase II complex	***POLR2G***	Pyrimidine Ribonucleotides Interconversion	37.72	5.27	32.45	−1	−0.25

		*Transcription elongation factors*					
Functional group	Gene name	Module	kTotal	kWithin	kOut	*log2* FC LPS	*log2* FC LPS+Etan
DSIF complex	*SUPT4H1*	NF-kB signaling	7.56	3.02	4.55	1.2	0.6
STAGA complex	*SUPT3H*	EIF2 signaling	2.64	0.73	1.92	−0.4	−0.03
SII class transcription elongation factor	*TCEA2*	Retinoate Biosynthesis I	60.06	4.29	55.77	−1	−0.5
	*TCEA3*	Regulation of IL-2 Expression in Activated and Anergic T Lymphocytes	12.89	5.27	7.62	−1.2	−0.6
	*TCEAL1*	Role of PKR in Interferon Induction and Antiviral Response	0.24	0.05	0.19	−0.2	−0.1
FACT complex	*SSRP1*	Retinoate Biosynthesis I	1.05	0.21	0.84	−0.5	−0.3

Genes within LPS-induced TNFα responsive co-expression modules possessing epigenetic regulation, transcriptional initiation and elongation properties. kTotal, total connectivity, k. kWithin, intra-modular connectivity. kOut, extra-modular connectivity. *log2* FC LPS, log2 transformed foldchange for the placebo-treated pre- and post-LPS challenged samples. *log2* FC LPS+Etan, log2 transformed foldchange for the etanercept-treated pre- and post-LPS challenged samples. Gene names marked in bold type denote module genes identified as top module hub genes.

### Specific epigenetic regulators, transcription initiation and elongation factors influenced by TNFα antagonism of the LPS-induced transcriptome

Induction of transcription is triggered by signal-dependent activation of DNA-binding transcription factors, accounting for the specific response to exogenous stimuli, which in turn recruit chromatin modifiers and RNA polymerase II [28]. Furthermore, these transcription factors can recruit histone-modifying enzymes such as histone acetyltransferases, deacetylases and methylases [29], which provide a landscape for signal-specific transcriptional initiation and elongation [30]. Thus, we inspected the TNFα responsive co-expression modules for epigenetic regulators, transcription initiation and elongation factors. **Table 3** lists those LPS-inducible epigenetic and transcription initiation/elongation factors that cluster in TNFα-responsive modules. Among these genes, *CBX7*, encoding chromobox homolog 7, and *POLR2G*, encoding the DNA-directed RNA polymerase II subunit RPB7, were also designated as hub genes for the role of PKR in interferon induction and antiviral response, and pyrimidine ribonucleotides interconversion co-expression modules, respectively (**Table 2**).

**Figure 4:**
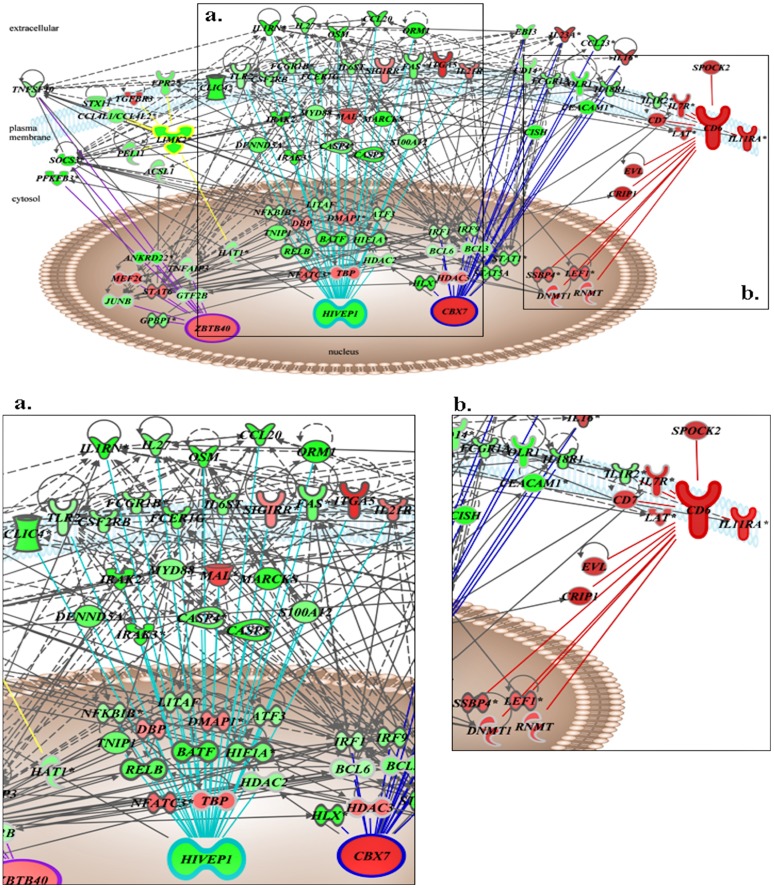
Interactome relationships of the core TNFα responsive co-expression modules. Integrating IPA (www.ingenuity.com) derived experimentally observed gene functional and co-expression network relationships allowed for the construction of a LPS-induced and TNFα-responsive gene activity model anchored at important hub genes. Panel **a**. illustrates the interactome relationships among transcripts in the NF-kB signaling and role of PKR in interferon induction and antiviral response modules with *HIVEP1* and *CBX7* as hub genes, respectively. Panel b. illustrates the interactome relationships among the regulation of IL-2 expression in activated and anergic T lymphocytes module with *CD6* as hub gene. Foldchanges (Red denotes high expression, green denotes low expression) derived from the differential gene expression analysis of the unpaired LPS+placebo and LPS+etanercept comparison where genes present significant ANOVA q-values (q<0.05). IPA interactome inference denoted by gray edges; gene coexpression network relationships denoted by turquoise (NF-kB signaling), purple (IL10 signaling), red (Regulation of IL-2 Expression in Activated and Anergic T Lymphocytes), yellow (Ephrin Receptor Signaling) and blue (Role of PKR in Interferon Induction and Antiviral Response) edges.

The NF-kB signaling, role of PKR in interferon induction and antiviral response, IL10 signaling, ephrin receptor signaling and regulation of IL-2 expression in activated and anergic T lymphocytes constitute core co-expression modules of the LPS-induced and TNFα-responsive transcriptome (**Figure 3A and B**). Integration of the IPA (Ingenuity® systems) further refined these core co-expression modules to a highly interconnected interactome network anchored at putative “driver” genes (module hubs) (**Figure 4**). This interactome is comprised of nuclear genes that include the methyltransferases *DNMT1* and *RNMT*, histone acetlyase/deacetlyases *HAT1*, *HDAC2* and *HDAC3*, as well as transcription factors that include *ATF3*, *IRF1*, *IRF9*, *NFKBIB*, *RELB*, *BCL6*, *BCL3*, *STAT1*, *STAT5A* and *STAT6*. Notably, *ATF3*, *HDAC2* and *STAT5A* exhibit pronounced differences in expression between the LPS response and etanercept treated LPS response (**Figure 4**). Cytosolic signaling molecules including *IRAK2*, *IRAK3*, *MARCKS* and the negative regulator of cytokine signaling *CISH* are prominent. Moreover, extracellular factors including *EBI3*, *CCL20*, *TNFAIP6* and the negative regulator of IL1 signaling, *IL1RN* are markedly influenced by etanercept treatment of the LPS response (**Figure 4**).

## Discussion

The majority of inducible transcriptional cascades are comprised of primary and secondary response genes. Stimulus-induced TNFα release constitutes a fundamental primary response in innate immunity, which leads to TNFα signal-specific secondary response cascades that encompass hundreds of genes. In the present study, we constructed a gene co-expression network for the human male systemic transcriptome induced by LPS challenge and identified those transcriptional modules influenced by TNFα inhibition. We demonstrate that the LPS-induced transcriptome is organized into functional modules enriched for genes involved in distinct biological themes exhibiting TNFα responsive and non-responsive elements. LPS-induced modules that were strongly influenced by TNFα inhibition include NF-kB signaling and pathways associated with T cell function, and further analyses suggested that TNFα antagonism inhibited transcriptional events in myeloid cells while enhancing transcription in lymphoid cells. In addition, we show that TNFα responsive modules are comprised of epigenetic regulators, transcription initiation and elongation factors. This study is the first to report on the role of TNFα in the systemic genomic response to an acute inflammatory stimulus in human males, using a controlled and reproducible model with relevance for acute inflammatory diseases [9]–[11].

Systems-level approaches, like the one we present in this study, will play an increasingly important role in defining the higher-order gene interactions that respond to exogenous and endogenous factors. Reductionist approaches alone limit the efficient interpretation of an overwhelming diversity in physiological responses to a stimulus, such as the response to the pro-inflammatory cytokine TNFα. Previous studies have documented TNFα dependence of certain systemic inflammatory responses, measured at protein level in plasma, induced by intravenous LPS administration in humans [12], [31], [32]. Since plasma TNFα levels peak 1.5–2 hours after LPS injection [12], [31], [32], and taking into account the dynamics of the genomic response to intravenous LPS [9], we chose the 4 hour post-LPS time point to evaluate the role of TNFα in the LPS-induced transcriptome in peripheral blood leukocytes. The co-expression network approach we adopted aided in constructing a scale-free leukocyte transcriptional network of pair-wise relationships in gene expression that respond in concert to LPS-induced TNFα release. Moreover, we leveraged the concepts of co-expression network hub genes, which are understood to be very important features of a gene co-expression network [21], [33]. In concordance with previous studies [9], upon LPS injection we observed a substantial reduction in nuclear, ribosomal and mitochondrial related processes, which include NRF2-mediated oxidative stress response, EIF2 signaling and mitochondrial dysfunction modules, respectively (**Figure 2**). Treatment with the TNFα inhibitor etanercept diminished, at least in part, the extent at which nuclear and ribosomal processes are reduced by LPS stimulation, whereas the mitochondrial pathway was not influenced by TNFα inhibition. Dysfunction in mitochondrial processes has been implicated in the severity and outcome of septic shock [34]. In addition, we show that genes involved in the regulation of T-cell activation present a significant reduction in transcript abundance after LPS challenge. TNFα inhibition dampens the LPS-induced reduction in transcription of genes in the regulation of IL-2 expression in activated and anergic T lymphocytes module (**Figure 2**), which include *CD6*, *IL7R*, *IL11RA* and *LAT* (**Figure 4**). Of note, the protein encoded by the *CD6* gene (cluster of differentiation 6) is a member of the scavenger receptor cysteine-rich superfamily [35], which was demonstrated to engage *E.coli* LPS [36]. Mice injected intraperitoneally with recombinant CD6 were protected from lethality due to endotoxemia and presented a reduction in serum TNFα abundance [36]. Our analyses categorized *CD6* as the T cell activation module hub gene. Disturbed lymphocyte function is a recognized hallmark feature of sepsis [37], [38]; our results suggest that these in part may be mediated by TNFα-mediated signaling. Mechanistically, these transcriptomic changes may arise due to altered TNF signaling within a cell therefore affecting the individual cells' transcriptional output, aberrant release of soluble mediators that respond to TNF in extravascular tissue and tissue-resident cells (for example, the liver Kupffer cell population), and differences in blood cell composition. Etanercept treatment had no significant effect on the cell counts and differentials (**Table 1**); thereby suggesting that the transcriptional changes were largely due to altered TNF signaling in individual circulatory cells and/or tissue-resident cells.

Transcription of genes within the NF-kB signaling module was highly increased after LPS-challenge and greatly influenced by TNFα inhibition (**Figure 2**). Central genes include *RELB*, encoding for a transcriptional factor that recognizes *kappab* promoter elements [39], and *IRAK3* (*IRAK-M*), a negative regulator of Toll-like receptor signaling [40], have been shown to be components of the cellular events that impart endotoxin tolerance [41], [42]. Endotoxin tolerance (or “leukocyte reprogramming”) is a typical property of circulating cells isolated from endotoxin challenged subjects or septic patients, characterized by a reduced capacity to release pro-inflammatory cytokines upon re-stimulation with LPS [43]. This immunosuppressive phenomenon has rightly received attention in developing new strategies for sepsis therapy [38], [44]. Our findings provide a network-based view of the transcriptional relationships that may conspire in immunosuppressive cellular states where the NF-kB signaling module hub gene *HIVEP1*, encoding human immunodeficiency virus type I enhancer binding protein 1, is predicted to be a highly influential (**Figure 4**). *HIVEP1* specifically recognizes and binds to the DNA sequence motif GGGACTTTCC within enhancer elements of both viral and host cellular genes suggesting a role in transcriptional regulation [45]. We predict *HIVEP1* as a LPS-induced and TNFα signal-specific modulator of the LPS-induced NF-kB signaling transcriptional module.

Transcriptional control of the LPS response is a highly ordered process of temporally distinct responses [11]. This involves a primary signal-specific (LPS-TLR4 axis) response (0.5–2 hrs) of post-translational modifications and subsequent nuclear translocation of key transcription factors including NF-kB. These primary response events are followed by a second wave of response genes [46]. Transcriptional control of these inducible genes has been linked to signal-dependent epigenetic modifications, transcriptional initiation and elongation [30]. Our findings provide evidence for TNFα signal-specific transcriptional modulation of a number of transcription initiation, elongation factors, as well as epigenetic regulators such as methyltransferases, deacetylases, chromodomain and bromodomain containing proteins (**Table 3**). The latter includes *BRD3*, encoding bromodomain containing 3, which participates in histone acetylation-dependant chromatin complexes; recently targeted by a potent immune suppressive histone mimic (termed I-BET), thereby highlighting its essentiality in selective inflammatory gene transcriptional programs [47]. Among the LPS-induced TNFα responsive epigenetic regulators *CBX7*, encoding the methylated histone residue binding transcriptional repressor chromobox homolog 7, was also identified as a co-expression module hub gene (**Table 2** and **3**; **Figure 4**). Our analysis further delineated peripheral transcripts, including the infectious disease susceptibility gene *CISH* [48], encoding cytokine inducible SH2-containing protein, and *EBI3*, encoding Epstein-Barr virus induced 3, putatively modulated by TNFα signaling (**Figure 4**). In the absence of significant differences in cell composition and differentials between our placebo- and etanercept-treated LPS-challenged subjects (**Table 1**), *CISH* and *EBI3* gene transcription is significantly down-regulated by TNFα inhibition of the LPS-induced response. Notably, EBI3, a subunit of IL-27 has been recently unmasked as a potential sepsis biomarker by genome-wide expression profiling [49]. Moreover, *EBI3* ^−/−^ mice were protected from sepsis-induced lethality by cecal ligation and puncture [50]. The impact of anti-TNF therapy on critically-ill septic patients presenting high serum IL-27 certainly warrants exploration.

Our study has limitations. The transcriptional cascades that ensue in whole-blood leukocytes post-LPS stimulation follow a highly ordered and temporally distinct path. Analyzing the genomic response to LPS stimulation and etanercept treatment solely at a 4-hour timepoint represents a portion of the leukocyte response; therefore, studies of the temporal properties governing the leukocyte transcriptional responses to LPS and TNFα antagonism is warranted. In addition to TNFα, etanercept has been reported to block lymphotoxin (TNFβ), which possibly limits the specificity of the observed effects.

Most knowledge of the role of endogenous TNFα during inflammatory disorders is derived from murine studies. Recent studies have underscored the relevance of the human endotoxemia model for investigating the genomic response in human inflammatory diseases [10], [11]. Importantly, while the genomic responses to different inflammatory stresses such as endotoxemia, burn and trauma are highly similar in humans, these responses are not reproduced in mice [11], further emphasizing the importance of dissecting the function of biological pathways such as those induced by TNFα in humans, rather than relying on mouse models. We here revealed an intriguing framework of a variety of biological and cellular pathways that are influenced by LPS-induced TNFα inhibition in humans *in vivo*. By combining genome-wide transcriptional profiling with the concepts of network biology we not only highlighted differentially expressed genes between comparisons but also defined co-expressed transcriptional networks anchored at module hub genes that predominantly possess transcriptional regulatory properties, including DNA binding, histone modifying and RNA polymerase II activity. These results provide comprehensive information about molecular pathways that might be targeted by therapeutic interventions that seek to inhibit TNFα activity during human inflammatory diseases.

## Supporting Information

Table S1 List of 4077 significant transcripts, their fold changes, *p*- and *q*-values.
(XLS)

File S1 Table S2: Quantitative PCR analysis primer pairs targeting transcripts encoded by *IL1RN*, *IRAK3*, *TNFAIP3*, *GPR84*, *MARCKS* and *B2M*. Figure S1: Quantitative PCR analysis results targeting transcripts encoded by *IL1RN*, *IRAK3*, *TNFAIP3*, *GPR84*, *MARCKS* normalized to *B2M*. *p<0.01 for saline (placebo) compared to LPS infusion (4 hours) by student *t* test. ^#^ p<0.05 for Etanercept-treated compared to Etanercept-treated LPS infused subjects (4 hours) by student *t* test.
(DOC)
