# Supplementary material for: Role of Tumor Necrosis Factor-α in the Human Systemic Endotoxin-Induced Transcriptome
Source: PLoS One. 2013 Nov 13;8(11):e79051. doi: 10.1371/journal.pone.0079051 (PMC3827317; doi:10.1371/journal.pone.0079051)
Supplement: File S1 — Table S2: Quantitative PCR analysis primer pairs targeting transcripts encoded by IL1RN, IRAK3, TNFAIP3, GPR84, MARCKS and B2M. Figure S1: Quantitative PCR analysis results targeting transcripts encoded by IL1RN, IRAK3, TNFAIP3, GPR84, MARCKS normalized to B2M. *p<0.01 for saline (placebo) compared to LPS infusion (4 hours) by student t test. # p<0.05 for Etanercept-treated compared to Etanercept-treated LPS infused subjects (4 hours) by student t test. (DOC) [file pone.0079051.s002.doc]

***Scicluna BP et al, Role of tumor necrosis factor-α in the human systemic endotoxin-induced transcriptome***

**Supporting Information**

**Table S2**

| **Primer name** | **Sequence 5' to 3'** |
| --- | --- |
| *GPR84* forward | CAGTGAGGGGATTTCATCTGAG |
| *GPR84* reverse | GCTCTTCTGGCTCCTTTAATTG |
| *IL1RN* forward | ATTGAGCCTCATGCTCTGTTCT |
| *IL1RN* reverse | ACTGTCTGAGCGGATGAAGG |
| *MARCKS* forward | GGTGCCCAGTTCTCCAAGAC |
| *MARCKS* reverse | CCTGCAGCTCCTCCTTGG |
| *TNFAIP3* forward | GAAGCACCATGTTTGAAGGAT |
| *TNFAIP3* reverse | AGGCCAGGATGTTCTTGCAG |
| *IRAK3* forward | GTACATCAGACAGGGGAAACTTT |
| *IRAK3* reverse | GACATGAATCCAGGCCTCTC |
| *B2M* forward | CTCGCGCTACTCTCTCTCTTTCT |
| *B2M* reverse | TGCTCCACTTTTTCAATTCTCT |

**Table S2.** List of qPCR primers used for technical validation of microarray data.

**Figure S1**

**
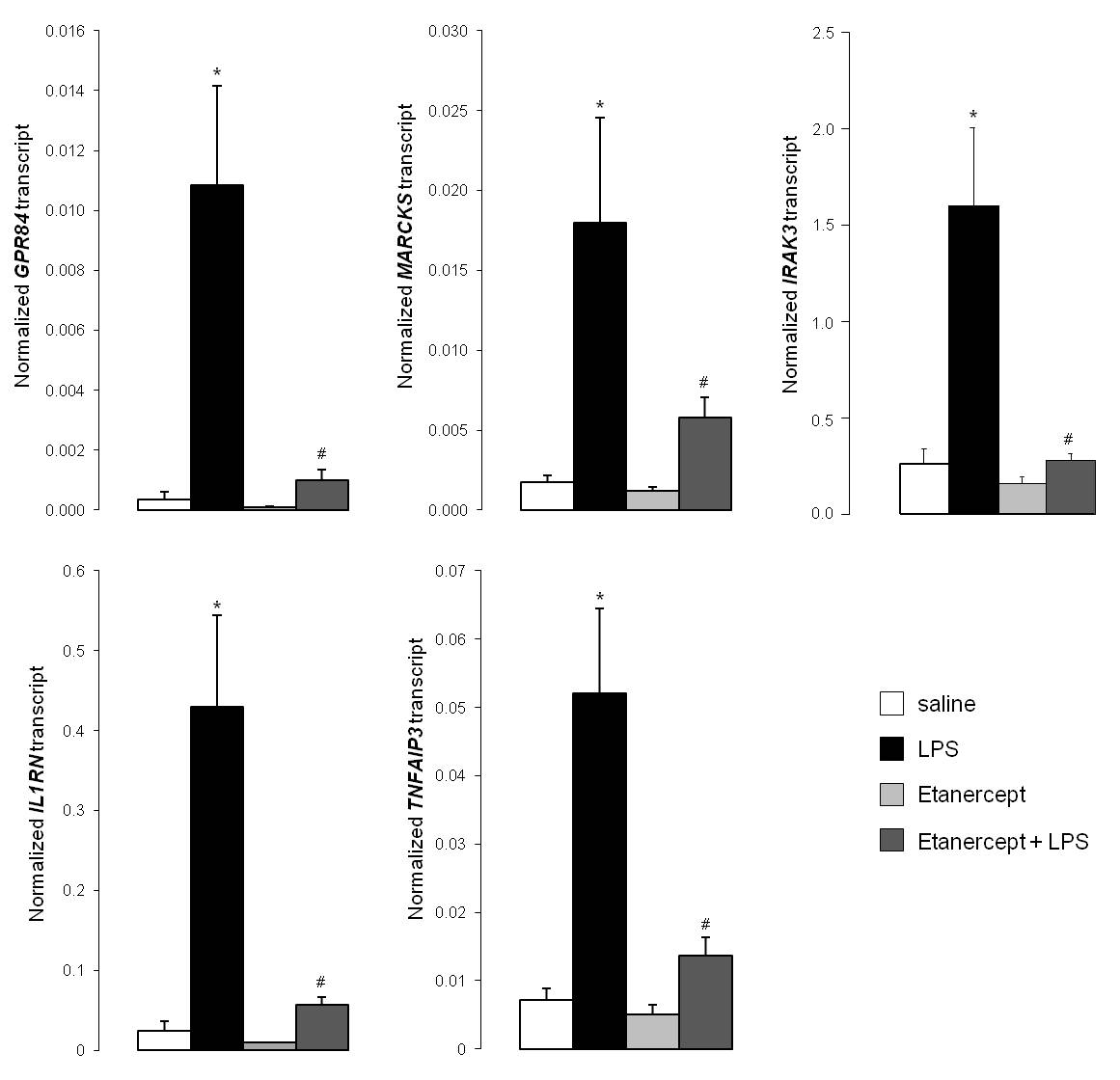
**

**Figure S1.** Technical validation of gene expression profiles by qPCR. Data was normalized to the *B2M* transcript. * p<0.001 between saline (placebo) and LPS (endotoxin) samples; ^#^p<0.01 between the LPS (endotoxin) and Etanercept-treated endotoxin response (Endotoxin+LPS).
